# Commissural misalignment independently predicts leaflet thrombosis after transcatheter aortic valve implantation

**DOI:** 10.1007/s00392-023-02192-6

**Published:** 2023-04-06

**Authors:** Susanne Jung, Fabian Ammon, Silvia Smolka, Maximilian Moshage, Mohamed Marwan, Stephan Achenbach

**Affiliations:** Medizinische Klinik 2, Kardiologie und Angiologie, Universitätsklinikum Erlangen, Friedrich-Alexander-Universität Erlangen-Nürnberg (FAU), Ulmenweg 18, 91054 Erlangen, Germany

**Keywords:** Commissural misalignment, HALT, Leaflet thrombosis, TAVI

## Abstract

**Aims:**

Transcatheter aortic valve implantation (TAVI) has become a minimally invasive alternative to surgical aortic valve replacement. Hypo-attenuated leaflet thickening (HALT)—a marker of subclinical leaflet thrombosis commonly detected by cardiac computed tomography (CT) after TAVI—may influence valve durability and function. The purpose of this study was to compare commissural alignment of the native and prosthetic aortic valves in cardiac CT in subjects with and without HALT and thereby identify commissural misalignment as potential predictor for leaflet thrombosis after TAVI.

**Methods and results:**

In 170 subjects, 85 with and 85 without HALT in post-TAVI CT, commissural orientation of the prosthesis was determined comparing native and prosthetic aortic valve orientation in cardiac CT by measuring the commissural angle relative to the right coronary ostium in the aortic valve plane. For the prosthetic valve, any deviation ≤ 15° compared to the native valve was classified as “aligned”; 16–30° as “mild”, 31–45° as “moderate” and ≥ 45° as “severe” misalignment.

Among subjects with HALT, median angular deviation was higher (36°, IQR 31°) than in the control group (29°, IQR 29°, *p = *0.042). “Severe” misalignment was more frequent in subjects who developed HALT (*n = *31, 37%) compared to the control group (*n = *17, 20%, *p = *0.013). In logistic regression analysis, more severe deviation (*p = *0.015, OR = 1.02 per 1° deviation) and “severe” misalignment (*p = *0.018, OR = 2.2) represented independent predictors for the occurrence of HALT after TAVI.

**Conclusion:**

Subclinical leaflet thrombosis after TAVI is associated with commissural misalignment. Potential clinical advantages of obtaining commissural alignment remain to be systematically assessed.

**Graphical abstract:**

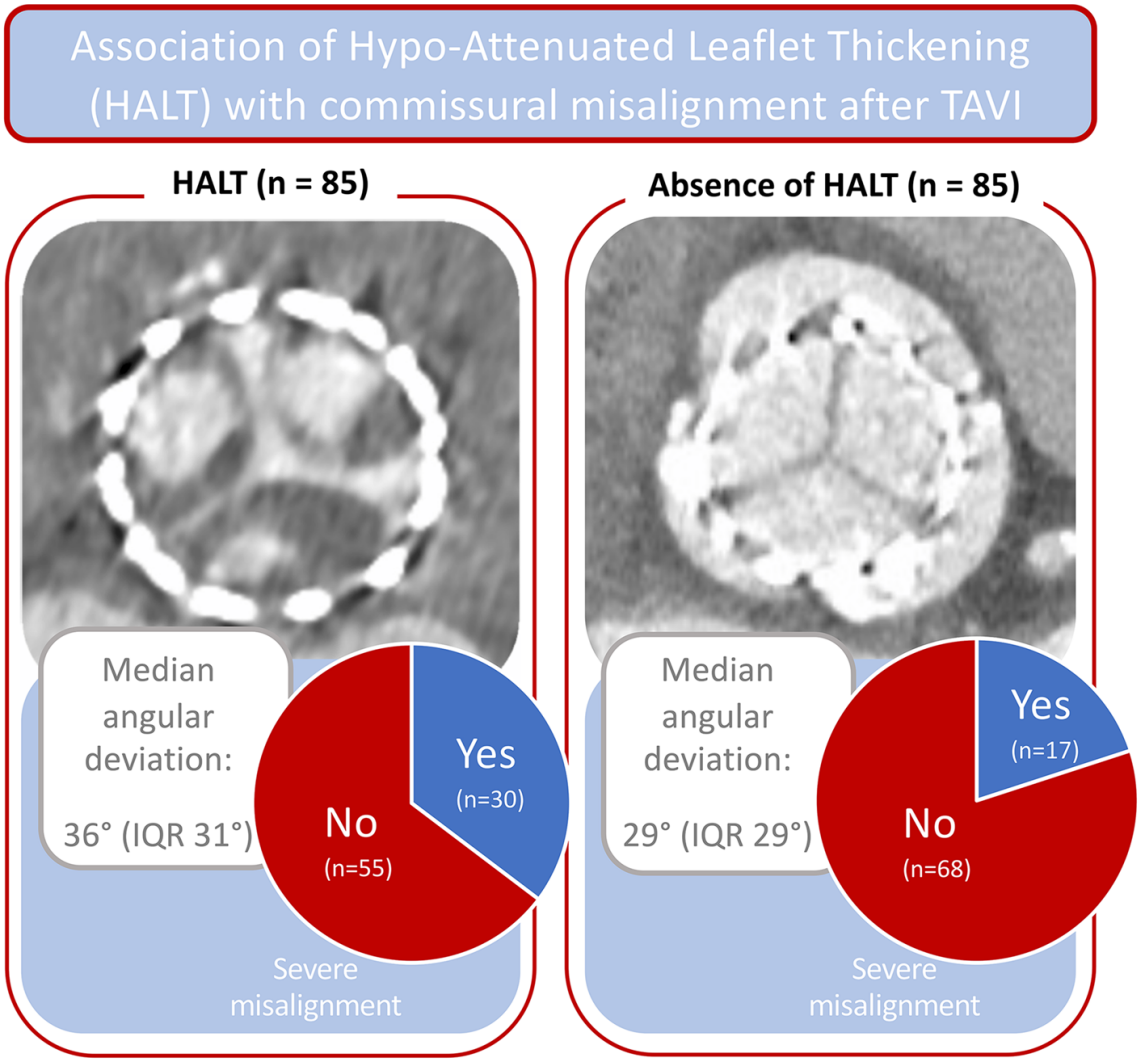

Association of HALT with commissural misalignment after TAVI. *HALT* hypo-attenuated leaflet thickening, *IQR* interquartile range, *TAVI* transfemoral aortic valve replacement

## Introduction

Transcatheter aortic valve implantation (TAVI) has become a minimally invasive alternative to surgical aortic valve replacement (SAVR) in subjects with high or intermediate surgical risk [1]. Increasingly, TAVI emerges as a valid treatment option for severe symptomatic aortic stenosis even in subjects with lower surgical risk [2–4]. With the indication of TAVI expanding towards subjects with lower risk profile and longer life expectancy, issues with respect to long-term durability and function of the implanted TAVI prosthesis gain increasing importance.

In this context, the influence of subclinical leaflet thrombosis after TAVI on valve durability and hemodynamics is currently under debate [5–7]. Hypo-attenuated leaflet thickening (HALT)—a marker of subclinical leaflet thrombosis commonly detected by cardiac computed tomography (CT) after TAVI—can be found in 10–20% of subjects undergoing TAVI or SAVR [8–12]. Subclinical leaflet thrombosis and reduced leaflet motion in bioprosthetic valves have been described to be associated with a higher incidence of transient ischemic attack and other thromboembolic complications [9, 13]. A recent publication by Garcia et al. even suggests an increased mortality in subjects with HALT compared to those without HALT after TAVI [10]. The elucidation of potential pathomechanisms favoring HALT after TAVI is, therefore, of major importance.

In contrast to SAVR, during which visual inspection allows the alignment of the bioprosthetic valve commissures with those of the native aortic valves, positioning of the bioprosthesis during TAVI usually occurs randomly and may lead to commissural misalignment [14]. Besides potentially causing overlap between the neocommissural posts and the coronary arteries, commissural misalignment has been shown to contribute to aortic regurgitation and leaflet stress [14–16].

We hypothesize that commissural misalignment, through altered flow patterns in the aortic root, may furthermore contribute to the occurrence of subclinical leaflet thrombosis. Therefore, the purpose of this study was to compare the commissural alignment of the native and prosthetic aortic valves in cardiac CT in subjects with and without HALT and thereby identify potential predictors for subclinical leaflet thrombosis after TAVI.

## Methods

### Study population

We present a retrospective analysis of a total of 170 patients—85 patients with HALT following TAVI and 85 controls. Study subjects were chosen from a cohort of 1200 patients who underwent transfemoral TAVI at our center between 2010 and 2020. Of these subjects, those who did not undergo cardiac CT after TAVI or who were lost to regular follow-up were excluded from further analysis. Of the remaining patients, those with HALT were chosen for our analysis and compared to a group of subjects who had also undergone CT after TAVI but had not demonstrated HALT. All of these patients suffered from severe symptomatic aortic valve stenosis and underwent cardiac CT before and after transfemoral TAVI at the Departments of Cardiology and Cardiac Surgery, University Hospital Erlangen, Germany. Subjects who underwent valve-in-valve TAVI were not included in this analysis. Severe aortic stenosis was defined according to 2021 European Society of Cardiology Guidelines for the Management of Valvular Heart Disease [1]. The indication for TAVI was established within the Heart Team. Written informed consent was obtained from each subject for the anonymous use of their data. Approval for retrospective evaluation of patient data for this study was obtained from the local ethics committee and the study was conducted according to the tenets of the Declaration of Helsinki and the principles of good clinical practice guidelines.

### Clinical parameters

Clinical data were assessed in each subject, including demographic data, medical history as well as type and size of the implanted prosthesis. For each subject, the STS (Society of Thoracic Surgeons) Score and logistic EuroSCORE (European System for Cardiac Operative Risk Evaluation) were determined. In addition, transthoracic echocardiography was performed in each subject before and after TAVI as well as during follow-up including the assessment of peak and mean pressure gradients (*p*_max_ and *p*_mean_) as well as peak velocity (*v*_max_) across the aortic valve.

### TAVI procedure

All subjects included in the analysis underwent transfemoral TAVI. The procedure was performed in a hybrid operating room under fluoroscopic guidance by a multidisciplinary team consisting of an anesthesiologist, cardiologists and cardiac surgeons. The procedure was either performed in general anesthesia or sedation, depending on the patient’s pre-procedural medical condition and operative risk. Selection of prosthesis type and size was based aortic root anatomy as determined by pre-procedural contrast-enhanced ECG-gated multidetector computed tomography.

### CT acquisition and assessment of commissural orientation in cardiac CT

**Fig. 1:**
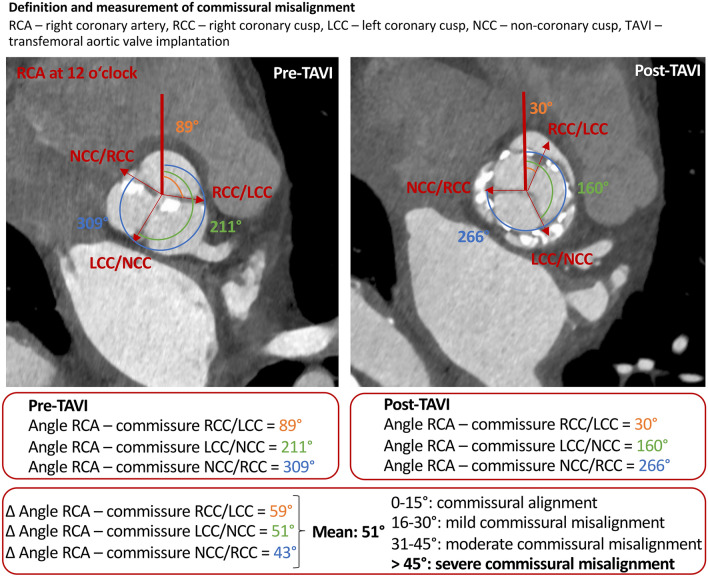
Definition and measurement of commissural misalignment. *RCA* right coronary artery, *RCC* right coronary cusp, *LCC* left coronary cusp, *NCC* non-coronary cusp, *TAVI* transfemoral aortic valve replacement

All subjects underwent dual-source CT scanning (Somatom Force, Siemens Healthineers, Erlangen, Germany) twice—up to 3 months before and within 12 months after TAVI. The subjects included in the analysis were non-consecutively selected from a group of patients, who, according to physician decision, received routine follow-up cardiac CT examination. Physician decision to perform a follow-up cardiac CT examination depended on the clinical status, symptoms, echocardiographic findings and renal function of the patients after TAVI. CT data acquisition was performed electrocardiogram-gated and contrast-enhanced according to a site-specific protocol for CT imaging in preparation for TAVI [17]. All cardiac CT scans were evaluated by the same experienced cardiologist who was blinded to all patient characteristics. HALT was defined as hypo-attenuated opacity attached to the valve affecting one or more leaflets evaluated in two-dimensional multiplanar reconstructions, as described previously [18–20]. Commissural orientation of the prosthesis was determined by comparing native (pre-TAVI scan) and prosthetic (post-TAVI scan) aortic valve orientation in cardiac CT using the software syngo.via (Siemens Healthineers, Erlangen, Germany) according to the method established by Fuchs et al. [14]. For this purpose, the orientations of the valve and prosthetic commissures were determined by measuring their angle relative to the right coronary artery (RCA) ostium in the aortic valve plane. This results in three angles—from the RCA ostium to the commissure between the right (RCC) and left coronary cusp (LCC), from the RCA ostium to the commissure between the LCC and non-coronary cusp (NCC) as well as from the RCA ostium to the commissure between the NCC and RCC. Deviations of these three angles (∆ angle) were determined comparing the angles in cardiac CT images before and after TAVI. Out of these three angular deviations, one mean angular deviation was finally calculated. For the prosthetic valve, a deviation ≤ 15° compared to the native valve was classified as “aligned”, 16°–30° as “mild” commissural misalignment, 31–45° as “moderate” and ≥ 45° as “severe” commissural misalignment (Fig. 1). This method was feasible in 100% of study subjects.

### Statistical analysis

All analyses were performed using SPSS software, version 21.0 (IBM Corporation, Chicago, IL, USA). First, distribution of data was assessed by Kolmogorov–Smirnov test and data were expressed as median (interquartile range, IQR) as they were not normally distributed. Not normally distributed metric data were compared using non-parametric tests for unpaired variables. A two-sided *p* value < 0.05 was considered statistically significant. For comparison of categorical variables, chi-square test or Fisher’s exact test for unpaired variables was used. Furthermore, logistic regression analysis was performed to assess potential predictors for HALT after TAVI.

## Results

### Clinical characteristics

Table 1 provides the clinical characteristics of the study groups. Median age in both groups was 81 (7) years (*p = *0.640). *N = *44 subjects (52%) in the HALT group and *n = *45 subjects (53%) in the control group were male (*p = *1.0). Logistic euroSCORE was 14 (13) in the HALT group and 12 (13) in the control group (*p = *0.248), STS-Score was 2 (2) in both groups (*p = *0.437).

**Table 1:** Clinical characteristics, intra- and post-procedural data of the study population

	HALT group (*n = *85)	Control group (*n = *85)	*p* value
Clinical characteristics			
Age [years]	81 (7)	81 (7)	0.640
Gender [male/female]	44/41	45/40	1.0
BMI [kg/m^2^]	28 (5)	27 (5)	0.063
Log. euroSCORE [%]	14 (13)	12 (13)	0.248
STS-Score [%]	2 (2)	2 (2)	0.437
LV-EF [%]	55 (9)	60 (5)	0.192
Oral anticoagulation	11 (13%)	17 (20%)	0.301
Intraprocedural data			
Category of prosthesis *n* (%)			
Balloon-expandable	40 (47%)	47 (55%)	0.357
Self-expandable	45 (53%)	38 (45%)	
Size of prosthesis *n* (%)			
23 mm	9 (10.6%)	7 (8.2%)	0.599
25 mm	8 (9.4%)	11 (12.9%)	0.465
26 mm	15 (17.6%)	21 (24.7%)	0.260
27 mm	23 (27.1%)	15 (17.6%)	0.141
29 mm	30 (35.3%)	31 (36.5%)	0.873
Postprocedural data			
*p*_max_ [mmHg]	16 (12)	16 (10)	0.469
*p*_mean_ [mmHg]	8 (7)	9 (5)	0.343
*v*_max_ [m/s]	2 (1)	2 (1)	0.373
Follow-up			
*p*_max_ [mmHg]	18 (14)	15 (10)	0.081
*p*_mean_ [mmHg]	9 (8)	8 (5)	0.067
*v*_max_ [m/s]	2 (1)	2 (1)	0.126

Data are given as median (interquartile range, IQR) or *n* (%)

*BMI* body mass index, *HALT* hypo-attenuated leaflet thickening, *LV-EF* left ventricular ejection fraction, *p*_*max*_ peak pressure gradient, *p*_*mean*_ mean pressure gradient, *STS* Society of Thoracic Surgeons,, *v*_*max*_ peak velocity

*N = *11 (13%) subjects from the HALT group and *n = *17 (20%) subjects from the control group were on oral anticoagulation (*p = *0.301). All other subjects received antiplatelet therapy. The number of subjects under anticoagulation or antiplatelet therapy did not differ significantly between subjects with and without HALT after TAVI (*p = *0.301). Left ventricular ejection fraction (LV-EF) was 55 (9) % in the HALT group and 60 (5) % in the control group (*p = *0.192).

In the HALT group, 40 subjects (47%) received a balloon-expandable and 45 (53%) a self-expandable valve. In the control group, 47 (55%) subjects received a balloon-expandable and 38 (45%) a self-expandable valve (*p = *0.179). Distribution of valve size did not differ significantly between the two groups (Table 1).

Postprocedural p_max_ and p_mean_ were 16 (12) and 8 (7) mmHg in the HALT group and 16 (10) and 9 (5) mmHg in the control group (*p = *0.469 and 0.343, respectively). *V*_max_ was 2 (1) m/s in both groups (*p = *0.373). Similarly, *p*_max_ and *p*_mean_ in the follow-up echocardiography did not differ significantly between the two groups (HALT group: 18 (14) and 9 (8) mmHg, control group: 15 (10) and 8 (5) mmHg, *p = *0.081 and 0.067, respectively). V_max_ in follow-up echocardiography was 2 (1) m/s (*p = *0.126).

### Commissural orientation

Median angular deviation was significantly higher in the HALT group (36 (31)°) compared to the control group (29 (29)°, *p = *0.042). In this context, the angular deviation of the RCA to the RCC/LCC commissure was significantly higher in subjects with HALT (38 (29)°) in comparison to those without HALT (28 (26)°, *p = *0.012). Angular deviation of the RCA to the LCC/NCC commissure was numerically higher in the HALT group (37 (42)°) than in the control group (30 (36)°), but did not reach statistical significance (*p = *0.108). Same was true for the angular deviation of the RCA to the NCC/RCC commissure (HALT group: 31 (35)°), control group: 29 (21)°), *p = *0.203).

Commissural alignment could be found significantly less often in subjects with HALT (*n = *13, 15%) than in subjects from the control group (*n = *23, 27%, *p = *0.045). However, there was no significant difference with respect to “mild” (HALT group: *n = *20, 24%; control group: *n = *23, 27%; *p = *0.362) or “moderate” commissural misalignment (both groups: *n = *22, 26%, *p = *0.569) in subjects with and without HALT. However, “severe” commissural misalignment could be detected more frequently in subjects with HALT (*n = *31, 37%) than in the control group (*n = *17, 29%, *p = *0.013). A comparison of commissural orientation between subjects with and without HALT after TAVI is given in Table 2.

**Table 2:** Comparison of commissural alignment between subjects with and without HALT after TAVI

	HALT (*n = *85)	No HALT (*n = *85)	*p* value
∆ Angles [°]			
Median ∆ angle	36 (31)	29 (29)	0.042
∆ Angle RCA–commissure RCC/LCC	38 (29)	28 (26)	0.012
∆ Angle RCA–commissure LCC/NCC	37 (42)	30 (36)	0.108
∆ Angle RCA–commissure NCC/RCC	31 (35)	29 (21)	0.203
Commissural (mis-)alignment			
Alignment	13 (15%)	23 (27%)	0.045
Mild commissural misalignment	20 (24%)	23 (27%)	0.362
Moderate commissural misalignment	22 (26%)	22 (26%)	0.569
Severe commissural misalignment	31 (37%)	17 (29%)	0.013

Data are given as median (interquartile range, IQR) or *n* (%)

*∆ angle* angular deviation, *HALT* hypo-attenuated leaflet thickening, *LCC* left coronary cusp, *NCC* non-coronary cusp, *RCA* right coronary artery, *RCC* right coronary cusp, *TAVI* transfemoral aortic valve implantation

### Predictors for HALT after TAVI

Logistic regression analysis was performed in order to identify potential predictors for HALT after TAVI (see Table 3). Univariate regression analysis revealed that echocardiographic parameters during follow-up, such as *p*_max_ (*p = *0.008, OR 1.05 per mmHg), *p*_mean_ (*p = *0.004, OR 1.11 per mmHg) and *v*_max_ (*p = *0.031, OR 2.3 per m/s) were significantly associated with leaflet thrombosis. Additionally, more severe median angular deviation (*p = *0.015, OR 1.02 per 1°), more severe deviation between the RCA and the RCC/LCC commissure (*p = *0.009, OR 1.02 per 1°) as well as between the RCA and the LCC/NCC commissure (*p = *0.044, OR 1.01 per 1°) could be identified as relevant predictors for HALT after TAVI. In this context, another relevant predictor was the occurrence of severe commissural misalignment (*p = *0.018, OR 2.3). In multivariate regression analysis, *p*_mean_ (*p = *0.009, OR 1.12) and severe commissural misalignment (*p = *0.010, OR 3.14) remained significant predictors for HALT after TAVI. In contrast, other parameters like size and type of implanted prosthesis (*p = *0.935 and *p = *0.283), body mass index (BMI, *p = *0.132) or anticoagulation regimen (*p = *0.851) were no significant predictors for HALT after TAVI.

**Table 3:** Predictors for the occurrence of HALT after TAVI

	*p* value	OR
Univariate regression analysis		
Median ∆ angle	0.015	1.021
∆ Angle RCA–commissure RCC/LCC	0.009	1.023
∆ Angle RCA–commissure LCC/NCC	0.044	1.013
Severe CMA	0.018	2.296
*p*_max_ follow-up	0.008	1.050
*p*_mean_ follow-up	0.004	1.109
*v*_max_ follow-up	0.031	2.275
Multivariate regression analysis		
*p*_mean_ follow-up	0.009	1.123
Severe commissural misalignment	0.010	3.140

*∆ angle* angular deviation, *HALT* hypo-attenuated leaflet thickening, *LCC* left coronary cusp, *NCC* non-coronary cusp, *OR* odds ratio, *p*_*max*_ peak pressure gradient, *p*_*mean*_ mean pressure gradient, *RCA* right coronary artery, *RCC* right coronary cusp, *TAVI* transfemoral aortic valve implantation, *v*_*max*_ peak velocity

## Discussion

In the current analysis, we compared the commissural alignment of the native and prosthetic aortic valves in cardiac CT in subjects with and without HALT after TAVI. There are three key findings: first, median angular deviation was higher in the HALT group compared to the control group, as was angular deviation of the RCA ostium to the RCC/LCC commissure. Second, “severe” commissural misalignment (defined as angular deviation between 45 and 60°) could be detected more frequently in subjects with HALT than in the control group, whereas commissural alignment was found less often in the HALT group. Third, high angular deviation and “severe” commissural misalignment were independent predictors for HALT after TAVI. Therefore, our results suggest that commissural misalignment may contribute to the occurrence of subclinical leaflet thrombosis after TAVI.

The findings of the current analysis are in accordance with those of a study by Khan et al. investigating anatomical characteristics associated with leaflet thrombosis after TAVI in a total of 167 subjects, from which 26 (15.6%) had HALT. There was a higher numerical incidence of commissural misalignment in subjects with HALT compared to those without HALT, but this did not reach statistical significance, possibly due to the low number of subjects with HALT [21]. Our analysis—including a higher number of subjects with HALT—was able to demonstrate a significantly higher incidence of commissural misalignment in subjects with HALT, thereby extending the results by Khan et al.

The findings of the current analysis can be interpreted in the context of Virchow’s triad. According to this theory, there are three factors contributing to the occurrence of thrombosis—blood constitution, endothelial dysfunction and alterations in blood flow patterns. Especially, the last two factors may explain the association between commissural misalignment and HALT described in the current analysis. With respect to endothelial dysfunction, Jilaihawi et al. suggested various factors contributing to delayed re-endothelialization, consecutively increasing the risk of leaflet thrombosis. These factors were stent frame expansion and fracture, implantation depth and symmetry as well as orientation of the native commissural and bioprosthetic leaflet [22]. Another factor that has been discussed to contribute to HALT after TAVI is overexpansion of the bioprosthetic stent frame, leading to endothelial injury and thereby increasing the risk of thrombus formation [23]. Accordingly, factors such as high angular deviation and severe commissural misalignment of the native and prosthetic valve may contribute to endothelial damage and delayed re-endothelialization, thereby increasing the risk of leaflet thrombosis. Regarding alterations in blood flow, data from cardiac magnetic resonance imaging after aortic valve sparing surgery for aortic regurgitation revealed that the formation of neo-sinuses between the native and bioprosthetic valve leaflets after TAVI contributes to increased blood stasis and thereby thrombus formation [24, 25]. Similarly, severe commissural misalignment may lead to altered flow patterns within the aortic root, resulting in increased blood stasis and thereby contributing to thrombus formation and HALT.

The current analysis offers a detailed examination of potential predictors for HALT after TAVI in cardiac CT. In the literature, several clinical and procedural contributors to HALT after TAVI have been described, such as the absence of oral anticoagulation, the use of a large prosthesis, moderate-to-severe paravalvular leakage or balloon-expandable valves [26–29]. The current analysis extends the knowledge about potential predictors for HALT after TAVI. In addition to clinical predictors for HALT after TAVI, such as *p*_max_, *p*_mean_ and *v*_max_ during follow-up examination, our analysis revealed severe commissural misalignment as an important independent predictor for HALT after TAVI in cardiac CT.

Currently, the development of feasible and safe TAVI implantation techniques in order to reach commissural alignment is an important topic in the literature [30]. Whereas in subjects undergoing SAVR, visual inspection allows the alignment of the bioprosthetic valve commissures with those of the native aortic valves, positioning of the prosthesis during TAVI occurs randomly and may lead to commissural misalignment [14]. Considering the results of the current analysis, an optimized implantation technique in order to allow commissural alignment might potentially contribute to the prevention of HALT after TAVI and thereby positively influence valve durability and functionality. In this context, a patient- and valve-specific, fluoroscopy-based implantation technique to obtain commissural alignment by rotation of the prosthesis at the level of the aortic valve has recently been published [30].

The current analysis has some limitations. First, its retrospective, single-center, cross-sectional design with a small sample size may impair statistical comparisons between the two groups. Second, the definition of four categories of commissural (mis-)alignment represents an arbitrary approach. However, this technique allows a comparable classification of commissural (mis-)alignment throughout the study population and has been applied successfully in other studies [14, 30]. Third, the results of the current study might possibly suggest, but do not prove an association between commissural misalignment and altered flow patterns in the aortic root, leading to HALT after TAVI. However, the current analysis was not designed in order to explore the pathophysiological, blood-flow related mechanisms behind the development of HALT, but to examine the role of commissural alignment in the development of leaflet thrombosis after TAVI. In this context, further exploration and comparison of aortic flow patterns in subjects with and without commissural misalignment after TAVI might offer interesting new insights into the pathophysiological mechanisms behind the occurrence of leaflet thrombosis. Similarly, the current analysis does not provide any information on clinical consequences of HALT, e.g. thromboembolic complications or clinical advantages of obtaining commissural alignment in subjects undergoing TAVI.

In conclusion, subclinical leaflet thrombosis after TAVI is associated with commissural misalignment. Pathophysiological, blood-flow-related mechanisms behind the development of HALT as well as a potential clinical benefit of attempting commissural alignment remain to be assessed.

## Data Availability

The data underlying this article will be shared on reasonable request to the corresponding author.

## Abbreviations

BMI: Body mass index
CT: Computed tomography
HALT: Hypo-attenuated leaflet thickening
IQR: Interquartile range
LCC: Left coronary cusp
LV-EF: Left ventricular ejection fraction
NCC: Non-coronary cusp
*p*_max_: Peak pressure gradient
*p*_mean_: Mean pressure gradient
RCA: Right coronary artery
RCC: Right coronary cusp
SAVR: Surgical aortic valve replacement
STS: Society of Thoracic Surgeons
TAVI: Transfemoral aortic valve implantation
*v*_max_: Peak velocity
